# The effects of a six-week exercise program on heart rate variability in adults with and without obstructive sleep apnea

**DOI:** 10.1007/s41105-026-00666-4

**Published:** 2026-07-06

**Authors:** Devon A. Dobrosielski, Stephen J. Ives, Karla A. Kubitz, Hyunjeong Park, Christopher Papandreou, Susheel Patil

**Affiliations:** 1 Department of Kinesiology, Towson University, Towson, MD 21252 USA; 2 Department of Health and Human Physiological Sciences, Skidmore College, Saratoga Springs, NY 12866 USA; 3 Department of Nursing, Towson University, Towson, MD 21252 USA; 4 Department of Nutrition and Dietetics Sciences, School of Health Sciences, Hellenic Mediterranean University, Siteia, Crete, Greece; 5 Clinical and Epidemiological Neuroscience, Institut d’Investigació Sanitària Pere Virgili (IISPV), Reus (Tarragona), Spain; 6 Division of Pulmonary, Critical Care and Sleep Medicine, University Hospitals, Cleveland, OH 44106 USA; 7 Case Western Reserve University, School of Medicine, Cleveland, OH 44106 USA

**Keywords:** Overweight, Obesity, Exercise training, Rehabilitation, Autonomic nervous system activity, Cardiac

## Abstract

**Purpose:**

Exercise training improves heart rate variability (HRV) and is recommended as a behavioral treatment option for obese adults suffering from obstructive sleep apnea (OSA). We examined whether the effects of a supervised exercise program on HRV parameters differed according to OSA severity in obese adults.

**Methods:**

Obese (BMI > 33 kg*m^− 2^) adults were evaluated for OSA using overnight polysomnography and subsequently classified as exhibiting no/mild OSA (AHI < 15 events*hour^− 1^) or moderate/severe OSA (AHI > 15 events*hour^− 1^). Cardiorespiratory fitness, body composition, and short-term heart rate variability (HRV) in frequency (VLF, LF, HF, LF/HF) and time (RMSSD, SDNN) domains were measured at baseline (Pre) and following a six-week (3 days/week) comprehensive exercise program (Post).

**Results:**

Twenty-seven adults (Age: 49 *±* 9 years; W:14; M:13) with no/mild OSA and 29 adults (Age: 52 *±* 9 years; W:15; M:14) with moderate/severe OSA completed all aspects of the trial. No significant differences in age, cardiorespiratory fitness, or HRV parameters were observed between groups at baseline. Across the sample, exercise training improved fitness (Pre: 30.5 ± 6.4 vs. Post: 32.4 ± 6.6 ml/kg/min, *p* < 0.001), VLF% (*p* = 0.015, η_p_^2^ = 0.11), and HF% (*p* = 0.016, η_p_^2^ = 0.11). An interaction of OSA severity and exercise training was observed for SDNN (interaction *p* = 0.01, η_p_^2^ = 0.12), such that the no/mild OSA increased HRV, whereas the moderate to severe OSA decreased HRV.

**Conclusion:**

These data suggest that while six weeks of exercise led to parasympathetic improvement across the sample, it was insufficient to improve sympathetic HRV signal among overweight adults with moderate to severe OSA.

## Introduction

Obstructive sleep apnea (OSA) is a chronic condition characterized by repeated episodes of partial or complete upper airway obstruction, with recurrent arousals and episodic oxyhemoglobin desaturation during sleep [1]. These pathological changes may alter the balance between sympathetic and parasympathetic nervous activity and, in turn, increase the incidence of cardiovascular disease (CVD) [2]. Various indices of heart rate variability (HRV), a non-invasive indicator of autonomic nervous system function, are clinically impaired in patients with OSA compared to healthy controls [3]. Yet, treatment of OSA with continuous positive airway pressure has not been found to reduce the rates of CVD-related events [4] or improve autonomic function (as determined by HRV) [5], in those with established OSA, underscoring the need for continued refinement of lifestyle management strategies aimed at lowering CVD risk in this clinical cohort.

To this end, regular exercise plays a significant role in reducing the risk of CVD [6], which may, in part, be explained by exercise-induced improvement in the activity of the autonomic nervous system. Indeed, Pichot et al. [7] reported improved nocturnal parasympathetic indices of HRV following 14 weeks of intensive aerobic exercise training in elderly men. After just eight weeks of regular endurance exercise, signs of increased parasympathetic tone at supine rest were observed in a cohort of post-acute myocardial infarction patients [8]. Also, an increase in parasympathetic activity and a decrease in sympathetic activity are observed in patients with type 2 diabetes mellitus (T2DM) after engaging in exercise interventions [9]. Importantly, the American Academy of Sleep Medicine recommends exercise as a behavioral treatment option for adults with OSA [10]. Since the publication of these guidelines, studies have documented improvements in HRV parameters in OSA patients following short-term (8 weeks) [11] and long-term (~ 40 weeks) [12] exercise training. Yet, this trend has not been observed across all studies. For example, while Berger et al. [13] noted a reduction in apnea-hypopnea index (AHI) and increased fitness in adults with moderate OSA who underwent 9 months of combined aerobic and resistance exercise training, the exercise intervention failed to improve nighttime HRV. Furthermore, Tonini et al. [14] exposed young, physically active adults to intermittent hypoxia while sleeping for 14 consecutive nights and subsequently observed an increase in diastolic blood pressure response during and a delayed recovery after a bout of submaximal exercise, reflecting an autonomic imbalance. Thus, these data suggest that the autonomic improvements conferred through regular exercise may be attenuated in the presence of OSA. Increasing our understanding of the relation between exercise and OSA may allow for improved behavioral strategies to reduce CVD risk in patients with and without OSA, especially with regard to lifestyle programs that include exercise recommendations.

Accordingly, the principal aim of this study was to investigate the effect of a six-week American College of Sports Medicine (ACSM) guided exercise program on HRV parameters in overweight adults with OSA. We hypothesized that exercise training would improve HRV, but improvements would be attenuated among those patients with moderate to severe OSA compared to those with mild or no OSA. To address this hypothesis, we measured resting short-term HRV parameters that are associated with increased risk of cardiac events and death [15, 16] and that can be improved with exercise training [11, 12].

## Materials and methods

Community volunteers were recruited to participate in a 6-week intervention to determine whether exercise training induced changes in markers of vascular structure and function varied according to OSA severity [ClinicalTrials.gov ID: NCT03219749]. The primary findings, recruitment procedures, and complete list of inclusion/exclusion criteria have been published elsewhere [17]. Briefly, overweight adults (body mass index (BMI): 27–42 kg/m^2^) between the ages of 30 and 65 years with a history of snoring were eligible to participate. Those individuals with a history of myocardial infarction or chronic heart failure, uncontrolled hypertension (SBP > 160 or DBP > 100 mmHg), type 1 or type 2 diabetes, or peripheral artery disease with claudication were excluded. Heart rate variability data were available on 56 volunteers who enrolled in and completed the intervention. The study was approved by the Towson University Institutional Review Board (Approval number: 16-A095), and all participants gave written informed consent prior to participation.

### Polysomnography assessment

Obstructive sleep apnea severity was assessed using overnight polysomnography during the baseline assessment prior to enrolling in the intervention. Standardized recording methods were used, including continuous monitoring (Alice 6, Philips-Respironics) as published elsewhere [17]. Sleep scoring criteria were established using the American Academy of Sleep Medicine scoring manual, Version 2.5. Hypopneas were counted if associated with 3% desaturation or arousal based on the recommended definition. Mean oxyhemoglobin saturation (Mean SpO_2_) during sleep, as well as total AHI, non-REM AHI and REM AHI was derived from the polysomnography. Total AHI was calculated as the number of apneas and hypopneas per hour of sleep. For the present analysis, total AHI (≥ 15 events/hour [i.e., moderate/severe] vs. <15 events/hour [i.e., no/mild]) was used to establish the OSA severity groups.

## Heart rate variability assessment

Cardiac autonomic function was estimated by resting HRV. Participants were tested between the hours of 06:00 and 09:00 am in a temperature-controlled (25 °C) laboratory following an overnight fast and 12 h after any moderate exercise. After approximately 50 min of supine rest during which primary outcome variables were being measured [17], a single-lead electrocardiogram (Dual Bio Amp, ADInstruments, Australia) was collected for five minutes at a sampling frequency of 1000 Hz and used to derive heart rate (HR). Participants were instructed to remain at rest and breathe normally throughout the five minutes of data acquisition, to ensure respiration was between 12 and 15 breaths per minute. The data were recorded and stored (Software LabChart 7.0, ADInstruments, Australia) for further analysis. Ectopic beats were removed to ensure analysis was conducted on validated R-R series free from artifact in both time and frequency domains. The study was designed to collect both time domain and frequency domain outcomes, as many reflect distinct dimensions of autonomic regulation and are influence by overlapping, yet distinct mechanisms [18].

The time domain analysis of HRV included mean and variance of all normal-to-normal R-R intervals, with the following calculated for the present study; (1) standard deviation (SDNN) and (2) coefficient of variation (CVRR) of all R to R intervals when ectopic beats are excluded, (3) percentage of successive R-R interals differing by more than 50 ms (pR50), and (4) squares of differences between adjacent R-R intervals (RMSSD).

The frequency domain analysis of HRV yielded the following parameters: (5) power in high frequency (HF; 0.15–0.40 Hz), (6) power in low frequency (LF; 0.04–0.15 Hz), (7) power in very low frequency (VLF; < 0.04 Hz), and (8) ratio LF (LF/HF). The presentation of the frequency-domain HRV data were expressed in relative terms (nu or %), in accordance with prior recommendations [19].

### Aerobic fitness assessment

Cardiorespiratory fitness was assessed at baseline and following the exercise intervention using a treadmill integrated with a Parvo Medics TrueOne 2400 metabolic measurement system (Parvo Medics, Salt Lake City, UT). All participants underwent a Bruce treadmill protocol and procedures for estimating maximal oxygen consumption have been outlined here [17].

### Body weight and composition assessment

Body mass index was calculated based on height and weight measures, which were derived using a stadiometer and digital scale, respectively. Body composition, namely total body fat percentage, was acquired using dual-energy X-ray absorptiometry (DXA) according to previously published procedures [17]. The Lunar Prodigy X-ray Bone Densitometer Scanner (GE Healthcare, Madison, WI) was calibrated on a daily basis according to manufacturer guidelines.

### Exercise intervention

American College of Sports Medicine guidelines were used to establish exercise training procedures for the 6-week intervention. Details regarding the exercise training prescription and progression are available here [17]. In general, participants attended three weekly sessions (18 total sessions) that were monitored by research assistants to ensure adherence to the prescribed exercise dosage and progression through the program. Each session consisted of approximately 40 minutes of aerobic training within an intensity of 60% to 90% of heart rate reserve, followed by 15–20 minutes of resistance exercises that involved 8–10 major muscle groups. Each session ended with a set of abdominal crunches and five minutes of a “cool down” on an aerobic training apparatus.

### Statistical analysis

Data were analyzed using open source software (JASP v0.95, Amsterdam, Netherlands). Assessment of baseline differences in characteristics and outcomes across OSA severity were determined using independent samples t-tests. To determine if OSA severity (no/mild vs. moderate/severe) differed in response to the exercise training program, two-way analysis of variance (ANOVA) was run on the HRV outcomes. Tests of assumption were performed for the baseline and ANOVA and if found, a non-parametric alternative was applied (baseline analysis) or an appropriate adjustment to the degrees of freedom was made (ANOVA). Statistical significance was established at *p* < 0.05, and estimates of effect size were provided to complement the p-values. Cohen’s D was used for the t-test, with 0.2, 0.5, and 0.8 interpreted as small, medium, and large effects, respectively. For the ANOVA, partial eta squared (η_p_^2^) with 0.01, 0.06, and 0.14 interpreted as small, medium, and large effects, respectively. Data are presented as means ± standard deviation unless noted otherwise.

## Results

### Participant characteristics

Patient characteristics at baseline are presented in Table 1. Patients across OSA groups were well-matched on age, height, weight, body composition, and aerobic fitness (all *p* > 0.05, d < 0.43, Table 1). By design, OSA severity differed by group.

**Table 1:** Baseline characteristics in patients with no/mild and moderate/severe OSA

Characteristics	No/Mild OSA	Moderate/severe OSA	*P* value
*Demographics*			
Age (years)	49 ± 9	52 ± 9	0.15
Female/Male	14/13	15/14	0.99^
Height (cm)	173 ± 9	171 ± 9	0.39
Weight (kg)	101 ± 18	100 ± 14	0.90
Body Mass Index (kg/m^2^)	33.5 ± 4.6	34.5 ± 4.3	0.45
*Sleep apnea parameters*			
AHI REM (events/hour)	19 ± 17	36 ± 23	0.05
AHI NREM (events/hour)	6 ± 3	28 ± 13	< 0.00
AHI Total (events/hour)	8 ± 4	29 ± 12	<0.00
Mean Oxygen Saturation (%)	95 ± 3	95 ± 1	0.42
*Cardiometabolic parameters*			
Total Body fat (%)	44.8 ± 6.0	45.9 ± 7.0	0.57
Systolic Blood Pressure (mmHg)	134 ± 12	135 ± 14	0.81
Diastolic Blood Pressure (mmHg)	82 ± 10	83 ± 8	0.72
VO_2_Peak (ml/kg/min)	32 ± 7	29 ± 6	0.17

Data are presented as mean ± SD

^Chi^2^contingency table testing

### Baseline HRV and OSA severity

HRV parameters at baseline were compared by OSA severity group and no significant differences were observed (all *p* > 0.05, Table 2). Time Domain and Relative Frequency Domain measures between the two groups were not significant; however HRV parameters trended to be higher in the moderate-severe OSA severity group (*p* < 0.10, d = 0.5).

### Responses to exercise training in OSA and the role of disease severity

Exercise training led to improvement across the sample in aerobic capacity (30.5 *±* 6.4 ml/kg/min vs. 32.4 *±* 6.6 ml/kg/min, (*p* < 0.001)) and total lean mass (54.5 *±* 11.4 kg vs. 55.0 *±* 11.1 kg; *p* = 0.04) and a reduction in total fat mass (43.1 *±* 8.5 kg vs. 41.9 *±* 8.5 kg; *p* < 0.001), although no differences in the change of these variables were noted between OSA severity groups (all *p*>0.05).

### Time domain HRV responses to exercise training in OSA and the role of disease severity

**Fig. 1:**
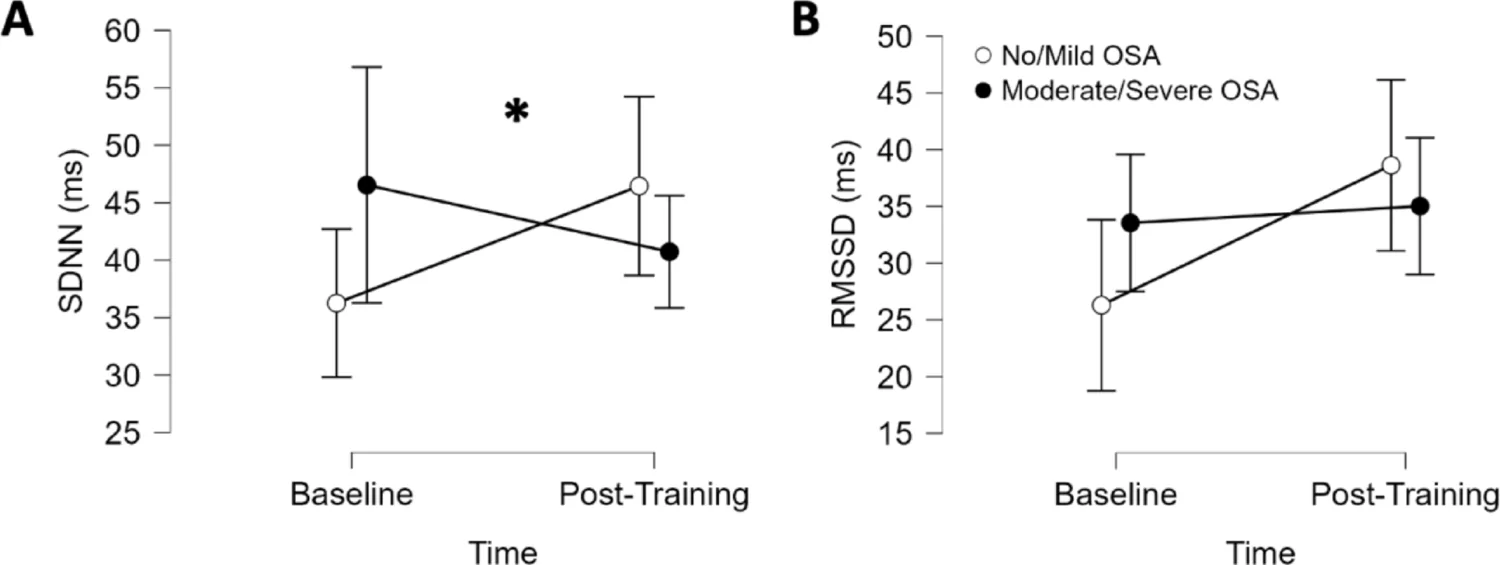
Heart rate variability responses to 6 weeks of exercise training in patients with no/mild and moderate/severe OSA. *SDNN* standard deviation of normal r-r intervals. *RMSSD* root mean square of successive differences between normal heartbeats. Data are means ± 95% Confidence Intervals. * *p* < 0.05, significant interaction of OSA severity and exercise training

All main effects and interactions for time domain HRV responses to exercise are presented in Table 2. No main effects for group or exercise training were detected for RMSSD or PR50. However, significant group-by-training interactions were observed for SDNN (interaction *p* = 0.01, η_p_^2^ = 0.12)(Fig. 1A) and CVRR (interaction *p* = 0.007, η_p_^2^ = 0.13), with increases in the no/mild OSA group and decreases in the moderate/severe group. RMSSD (Fig. 1B) showed no main effects or interactions (all, *p* > 0.05).

### Frequency domain HRV Responses to exercise training in OSA and the role of disease severity

**Fig. 2:**
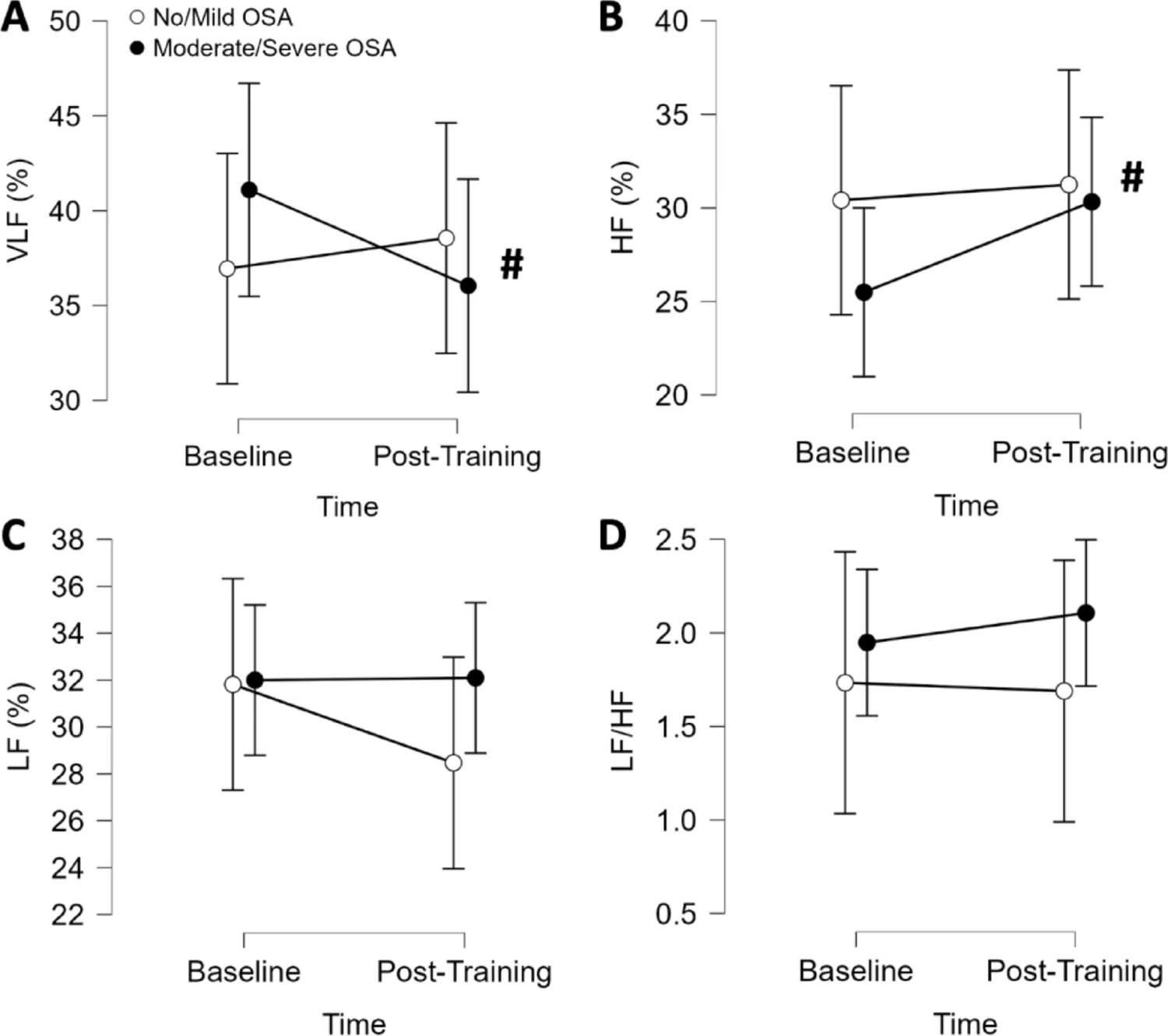
Heart rate variability responses to 6 weeks of exercise training in patients with no/mild and moderate/severe OSA. *VLF%* relative very low frequency, *HF%* relative high frequency, *LF%* relative low frequency, *LF/HF* ratio of LF to HF power. Data are means ± 95% Confidence Intervals. # *p* < 0.05, significant main effect of exercise training

All main effects and interactions for frequency domain HRV responses to exercise are presented in Table 2. A significant increase in VLF% (*p* = 0.015, η_p_^2^ = 0.11) (Fig. 2A) and HF% (*p* = 0.016, η_p_^2^ = 0.11) (Fig. 2B) was observed following exercise training. However, there were no main effects of group or interaction effects for VLF% and HF% (all *p* > 0.05). Analysis of LF% and the LF/HF ratio, revealed no main effects of group or exercise training (Fig. 2C and D). The analysis of the absolute frequency domain parameters for LF and HF revealed no main effects of OSA severity or exercise training (all, *p* > 0.05, data not shown). Although no other main effects were observed for group (*p* = 0.29, η_p_^2^ = 0.02) or exercise training (*p* = 0.11, η_p_^2^ = 0.05) in absolute VLF power, it neared significance on the interaction of group and exercise training (interaction *p* = 0.05, η_p_^2^ = 0.07), documenting a similar pattern described above.

**Table 2:** HRV at baseline and following 6-weeks of exercise training in patients with no/mild and moderate/severe OSA

	Baseline		6-weeks				
	No/Mild OSA	Moderate/ Severe OSA	No/Mild OSA	Moderate/ Severe OSA	OSA severity main effect	Time main effect	OSA severity x time interaction
Heart rate (bpm)	65 ± 7	65 ± 6	63 ± 6	63 ± 6	0.769	0.519	0.705
*HRV time domain measures *							
RMSSD (ms)	26.3 ± 11.5	33.5 ± 27.0	38.6 ± 29.1	35.0 ± 17.4	0.718	0.679	0.105
CVRR (%)	0.04 ± 0.02	0.05 ± 0.03	0.05 ± 0.02	0.04 ± 0.01	0.614	0.381	0.007
SDNN (ms)	36.3 ± 16.3	46.5 ± 27.0	46.5 ± 19.7	40.7 ± 12.9	0.593	0.248	0.01
PR50 (%)	8.0 ± 8.6	9.0 ± 7.1	17.3 ± 20.6	16.6 ± 17.8	0.957	0.481	0.702
*Relative HRV frequency domain measures*							
VLF (%)	36.9 ± 16.6	41.1 ± 18.4	38.6 ± 17.1	36.0 ± 15.3	0.817	0.015	0.226
LF (%)	31.8 ± 11.4	32.0 ± 13.0	28.5 ± 12.5	32.1 ± 15.2	0.463	0.883	0.369
HF (%)	30.4 ± 16.6	25.5 ± 13.0	31.2 ± 18.3	30.3 ± 19.1	0.414	0.016	0.426
LF/HF (%)	1.7 ± 1.8	2.0 ± 1.4	1.7 ± 1.7	2.1 ± 2.4	0.428	0.728	0.712

Data are presented as mean ± SD

## Discussion

The present study examined whether six weeks of aerobic exercise, combined with resistance training, improved HRV parameters in a cohort of obese adults with and without OSA. We found that while exercise led to improvements in aerobic capacity and body composition, no changes in time domain HRV (e.g., RMSSD) were observed. To the contrary, frequency domain HRV (i.e., HF%) was higher at follow-up, suggesting an improvement in parasympathetic activity following exercise training, regardless of OSA severity. Finally, we noted an interaction based on OSA status, such that SDNN improved among those with no/mild OSA, whereas SDNN was reduced among those with moderate/severe OSA.

Numerous studies have reported an improvement in OSA-related outcomes with exercise training, yet only three previous investigations [11–13] have examined the effects of exercise programming on HRV parameters among those with OSA. In a seminal report by Berger et al. [13], the authors examined HRV during sleep before and after randomizing 74 OSA patients (age: 40–80 years; AHI: 15–30 events/hour) into a 9 month combined resistance and aerobic exercise program (3 days per week) or a control group that received diet and physical activity advice. SDNN, RMSSD, and HF power worsened (-7.8%, -10.3% and − 24%, respectively) in the control group, but were maintained in exercisers. Cipriano et al. [11] examined the impact of aerobic exercise (3 days per week) vs. CPAP therapy (*≥* 4 h per night; 70% of nights) for 8 weeks on short-term (10 min at rest) HRV parameters among 39 OSA patients (*≥* 15 AHI) with and without hypertension. They found that exercise training improved HF and LF values and, in turn, the LF/HF ratio, while no change in these parameters was observed in the group that was prescribed CPAP. Importantly, the improvement in LF/HF with exercise was found in those with and without hypertension. More recently, Araujo and colleagues [12] reported greater positive changes in SDNN and RMSSD (5 min at rest), albeit no changes in frequency domain parameters, in 16 OSA patients (age: 40–65 years; AHI *≥* 15 events/hour) following the completion of an exercise trial (3 days per week for 40 weeks of combined training) compared to sedentary controls. Collectively, these studies suggest that exercise training favorably influences cardiac autonomic modulation in patients suffering from OSA. In consideration of this general trend, the present study adds to the literature in the following ways.

Our intervention was shorter than those cited above. The a priori decision to employ a six-week exercise program was based on our hypothesis that improvements in nitric oxide-mediated endothelial function of the brachial artery, the primary outcome of the larger parent study [17], would occur as early as 4 weeks from beginning the trial. Consequently, our data extend previous findings by revealing a potentially rapid time course of exercise-induced adaptations to cardiac autonomic modulation in obese adults, most of whom suffer with mild to severe OSA. Specifically, the changes in HF and VLF (which is not affected by sympathetic blockade [18]) suggest that any improvement in autonomic activity for those with OSA in the short term may be largely dependent on parasympathetic reactivation. This point is underscored by the fact that we observed a significant increase in aerobic capacity, which is associated with increased cardiac vagal tone. Still, we acknowledge that RMSSD, the primary time domain measure used to estimate vagally mediated changes reflected in HRV [20], was not altered after 6 weeks of training. Further, the HF band, or respiratory band, corresponds to variations in heart rate related to the respiratory cycle and therefore indexes vagal modulation of heart rate, but it may not provide a pure index of cardiac vagal tone [21]. Though it might be worth noting that there were no reductions in any marker of parasympathetic activity, as observed in the control by Berger et al. [13] suggesting that exercise training may slow the deterioration of ANS activity in individuals with OSA. Given the modest changes in response to the exercise training in the current study, it is possible that OSA attenuates the cardiac autonomic adaptations to exercise training, or that further training intensity or volume are needed to elicit more robust changes and warrants further study.

The inclusion of overweight particiants with no or mild OSA to the parent project [17] is a novel design choice based, in part, on the work by Borel et al. [22] who reported that weight loss and improvements in select cardiometabolic risk factors with a lifestyle intervention program were attenuated in men suffering from severe OSA compared to weight matched men without OSA. Related to the current topic, others [14] have shown in healthy men that exposure to 14 consecutive nights of intermittent hypoxic (a cardinal symptom of OSA) results in a greater increase in diastolic blood pressure during submaximal exercise, and a longer recovery from exercise, inferring that intermittent hypoxia is the main stimulus inducing cardiovascular autonomic imbalance. We, in turn, reasoned that regular exercise may not serve as an effective countermeasure to the detrimental effects of OSA on HRV, and subsequently divided our sample to reflect differences in OSA severity. In line with this reasoning, we observed several time domain HRV parameters, notably SDNN, were improved with exercise training in the group with no or mild OSA (AHI < 15 events/hour), but worsened in the group with moderate to severe OSA (AHI *≥* 15 events/hour).

The mechanisms underlying these disparate responses are not immediately clear, but it is important to note that while the variation in SDNN in short-term HRV recordings is primarly influenced by the parasympathetically-mediated respiratory sinus arrhythmia (RSA) [20], sympathetic nervous system activity also plays a role [23]. In OSA, chronic exposure to intermittent hypoxia increases the excitability of carotid chemoreceptors, thereby increasing the activity of the afferent fibers serving the sympathetic vasomotor centers in the brainstem and ultimately resulting in increased sympathetic tone [24]. The hyperexcitability of chemoreceptors can, in turn, attenuate baroreflex function at the level of the central nervous system [11]. Alternatively, baroreflex control in OSA may be impaired secondary to decreased compliance in the carotid sinuses and aortic arch. Indeed, increased arterial stiffness is observed in OSA patients [25], and we failed to observe improvements in pulse wave velocity, a marker of arterial stiffness, following six weeks of exercise [17]. All that being stated, it is reasonable to infer that six weeks of exercise training is not long enough to overcome the burden of continuous episodes of chronic intermittent hypoxia on the SNS or alter vascular stiffness.

Whether SDNN would have continued to decline had we extended our intervention beyond six weeks is unknown and a limitation. However, this seems unlikely considering that Araujo and colleagues [12] employed a similar exercise protocol for 40 weeks in OSA subjects with AHI *≥* 15 and found significant improvements in SDNN, as well as favorable changes in AHI (-5 events/hour) and O_2_ desaturations (-3 events/hour). Further, others [26] have confirmed, using microneurography, that sympathetic nerve activity during isometric handgrip exercise is improved in OSA patients (AHI *≥* 15 events/hour) following 72 sessions (~ 24 weeks) of aerobic combined with resistance exercise that also reduced AHI (− 6 events/hour). Thus, clinically meaningful improvement in sympathetic autoregulation with exercise training may occur only when training is sufficient in duration and/or OSA severity is reduced to a certain threshold or is resolved completely. Clearly, parceling out whether exercise influences arterial baroreflex control or chemoreflex hypersensitivity independent of changes in OSA severity requires a longer follow-up with a more robust methodological approach.

We remain cautious in our interpretation of our findings upon reflection of various limitations. Polysomnography testing following the intervention was not possible, therefore, whether changes in HRV are associated with improvements in OSA severity cannot be determined. Polysomnography data were collected to evaluate the severity of OSA at baseline and not to determine the efficacy of the exercise for improving the disorder. Second, while we propose that chronic intermittent hypoxia is the primary mechanism responsible for the modulation of HRV we used an AHI threshold of 15 events/hour to distinguish between those with more severe vs. less severe OSA. Yet, recent data[27] reveal the shortcomings of using conventional metrics (e.g., AHI) in lieu of emerging metrics (e.g., hypoxic burden [28]) to define OSA severity and, in turn, to predict cardiovascular risk [29] and responsiveness to treatment. However, exploration of the most clinically meaningful OSA phenotype lies beyond the scope of the current paper. Third, we were equipped to measure short-term HRV, but recognize that 24-hour HRV recordings remain the “gold standard” due to their ability to reflect circadian rhythms, core body temperature, metabolism, the sleep cycle, and the renin-angiotensin system [20]. We further recognized that sympathetic activity is best captured through the assessment of muscle sympathetic nerve activity [26]. Fourth, we emphasize that HRV was a secondary outcome within a parent trial, and therefore was not designed to detect small, to moderate, changes in autonomic function [17]. Therefore, our present findings of significant interaction by OSA status for SDNN, but no main effect for time or OSA severity group, may be the result of insufficient power. To this point, the range of HRV values were large and may have masked the main effect in the absence of a much larger sample size. Fifth, we acknowledge the risk of spurious significant findings associated with multiple comparisons but also recognize that Bonferroni correction is likely to increase the type II error rate. Moreover, any adjustment to the alpha will not change the effect size, which we have reported herein. Nevertheless, the various strengths of the study include the recruitment of apparently healthy, community-dwelling adults without prior OSA diagnosis, the implementation of a comprehensive, ACSM guidelines-driven exercise intervention, and the use of validated parameters of HRV.

## Conclusion

The current study sought to examine the effects of six weeks of combined aerobic and resistance training on HRV in a cohort of obese adults with moderate-severe OSA versus no-mild OSA. We observed no changes in RMSSD, a keystone time-domain HRV parameter, despite significant improvements in aerobic fitness and body composition. In the frequency domain, we observed a higher HF% following training, independent of OSA severity, suggesting an improvement in parasympathetic activity following the intervention. In addition, we noted an interaction based on OSA status, such that SDNN improved among those with no/mild OSA, whereas SDNN was reduced among those with moderate/severe OSA. Collectively, these current findings suggest that six weeks of concurrent training may improve frequency-based measures of parasympathetic dominant HRV (e.g., HF). More work is needed, using larger samples, longer and/or more intensive exercise protocols (e.g., high intensity interval training), and/or exploration of HRV-guided exercise training to better ascertain the possible influence of exercise training on individuals with OSA.
